# Adaptive truncation of the S gene in IBV during chicken embryo passaging plays a crucial role in its attenuation

**DOI:** 10.1371/journal.ppat.1012415

**Published:** 2024-07-30

**Authors:** Rong Liang, Kangchengyin Liu, Yingfei Li, Xuehui Zhang, Linqing Duan, Min Huang, Lu Sun, Fang Yuan, Jing Zhao, Ye Zhao, Guozhong Zhang

**Affiliations:** 1 National Key Laboratory of Veterinary Public Health Security, College of Veterinary Medicine, China Agricultural University, Beijing, China; 2 Key Laboratory of Animal Epidemiology of the Ministry of Agriculture, College of Veterinary Medicine, China Agricultural University, Beijing, China; KU: The University of Kansas, UNITED STATES OF AMERICA

## Abstract

Like all coronaviruses, infectious bronchitis virus, the causative agent of infectious bronchitis in chickens, exhibits a high mutation rate. Adaptive mutations that arise during the production of live attenuated vaccines against IBV often decrease virulence. The specific impact of these mutations on viral pathogenicity, however, has not been fully elucidated. In this study, we identified a mutation at the 3’ end of the S gene in an IBV strain that was serially passaged in chicken embryos, and showed that this mutation resulted in a 9-aa truncation of the cytoplasmic tail (CT) of the S protein. This phenomenon of CT truncation has previously been observed in the production of attenuated vaccines against other coronaviruses such as the porcine epidemic diarrhea virus. We next discovered that the 9-aa truncation in the S protein CT resulted in the loss of the endoplasmic-reticulum-retention signal (KKSV). Rescue experiments with recombinant viruses confirmed that the deletion of the KKSV motif impaired the localization of the S protein to the endoplasmic-reticulum-Golgi intermediate compartment (ERGIC) and increased its expression on the cell surface. This significantly reduced the incorporation of the S protein into viral particles, impaired early subgenomic RNA and protein synthesis, and ultimately reduced viral invasion efficiency in CEK cells. In vivo experiments in chickens confirmed the reduced pathogenicity of the mutant IBV strains. Additionally, we showed that the adaptive mutation altered the TRS-B of ORF3 and impacted the transcriptional regulation of this gene. Our findings underscore the significance of this adaptive mutation in the attenuation of IBV infection and provide a novel strategy for the development of live attenuated IBV vaccines.

## Author summary

The administration of live attenuated vaccines is currently recognized as the most effective strategy for preventing infections caused by IBV. Understanding how mutations arising during vaccine production influence IBV virulence is crucial for developing more effective vaccines. In this study, we focused on a specific mutation in the S gene of IBV, which arose during viral propagation in chicken embryos. This mutation resulted in the truncation of the CT region of the S protein and concurrently eliminated a critical motif required for the retention of the protein at the viral assembly site. Similar S protein truncation has been observed in the production of attenuated vaccines against other coronaviruses, suggesting that the mutation identified in IBV in the present study was an adaptive mutation driven by natural selection pressure. Our findings underscore the value of targeting this adaptive mutation site as a strategy for reducing IBV virulence, and for the development of effective live attenuated vaccines against IBV and other coronaviruses.

## Introduction

Coronaviruses (CoVs), which belong to the *Coronaviridae* family within the order *Nidovirales*, are a group of positive-sense RNA viruses that can infect a wide range of host species [1–4]. CoV can be further classified into four genera: α-CoV, β-CoV, γ-CoV, and δ-CoV. Infectious bronchitis virus (IBV) is a member of the γ-CoV genus and causes widespread infections in commercial chicken populations worldwide, resulting in significant economic losses [5,6]. IBV shares structural similarities with other well-known CoVs such as porcine epidemic diarrhea virus (PEDV), severe acute respiratory syndrome CoV 2 (SARS-CoV-2), and mouse hepatitis virus (MHV) [7–9]. Mature virions of these viruses are composed of four structural proteins: the spike (S), envelope (E), membrane (M), and nucleocapsid (N) proteins [10,11]. A fifth structural protein, the hemagglutinin-esterase (HE), is present in a subset of β-coronaviruses [12]. Additionally, different CoVs encode unique group-specific accessory proteins that play various roles in the viral lifecycle; in IBV, these include proteins such as 3a, 3b, 5a, and 5b [13–15]. The coronavirus exhibits an enormous capacity to mutate, driven by both spontaneous mutations and genetic recombination [16]. This mutation rate is further increased by a RNA-dependent RNA polymerase (RdRp) which lacks proofreading ability [17]. Another crucial mechanism leading to CoV mutation is homologous RNA recombination; this process is triggered by template switching, which is facilitated by IBV’s unique discontinuous transcription mechanism [18,19].

Currently, the administration of live attenuated vaccines is considered the most effective strategy for preventing IBV infection [20,21]. These vaccines are typically produced by serially passaging the virus in embryonated chicken eggs (ECEs). Due to the high mutation rates of CoVs, adaptive mutations frequently arise during the vaccine production process [22]. These mutations are often associated with a reduction in virulence, which is crucial for the safety and efficacy of live attenuated vaccines. In the present study, during the serial passage of an isolated IBV strain in embryonated chicken eggs, we identified a nucleotide mutation (G to T) at the 3’ end of the S gene. This mutation converted a glutamic acid at position 1,159 in the S gene to a stop codon, which truncated the cytoplasmic tail (CT) domain of the S protein by removing nine amino acids (EQYRPKKSV). Similar truncating mutations in the CT of the S protein have also been observed in other CoVs, such as PEDV and swine acute diarrhea syndrome CoV (SADS-CoV) [23,24]. Intriguingly, analogous mutations resulting in the premature termination and consequent truncation of the CT have been identified in the glycoproteins of RNA viruses, including human immunodeficiency virus-1 (HIV-1), simian immunodeficiency virus (SIV), and equine infectious anemia virus (EIAV) [25–28]. These truncations are associated with a decrease in viral replication and pathogenicity in vivo. Notably, the endoplasmic reticulum retention motif (ERRS) is concurrently absent from the site of the 9-aa truncation in the IBV. The CoV ERRS motif, which can be present in either the dilysine or dibasic form (KxKxx, KKxx, or KxHxx, where x represents any aa residues), serves as a weak ER-Golgi intermediate compartment (ERGIC) retention signal [29,30]. Its involvement in protein transport and intracellular accumulation is crucial for the efficient assembly of CoV particles [23,29]. Additionally, the G to T mutation is particularly significant as it occurs within the transcription regulatory sequence-body (TRS-B) of the open reading frame 3 (ORF3), altering the TRS-B from CTGAACAA to CTTAACAA. This change aligns the TRS-B with the TRS-leader (TRS-L), potentially impacting the transcriptional regulation of viral genes. However, the mechanism by which this mutation site influences viral virulence remains poorly understood.

In this study, we confirmed the widespread presence of an adaptive G to T mutation at the 3’ end of the S gene across various IBV strains, and demonstrated that it was critical for reducing IBV virulence. Further investigation into the virulence attenuation mechanism revealed that the mutation led to the deletion of the ERRS motif in the S protein, impairing its proper localization to the viral assembly site in the ERGIC. This mislocalization significantly reduced the incorporation of the S protein into viral particles, thereby decreasing IBV’s ability to enter host cells. Overall, our findings provide a novel strategy for the development of attenuated live vaccines against IBV.

## Results

### Mutation of GAA to TAA in the S gene is a common feature in chicken embryo-attenuated IBVs

**Fig 1:**
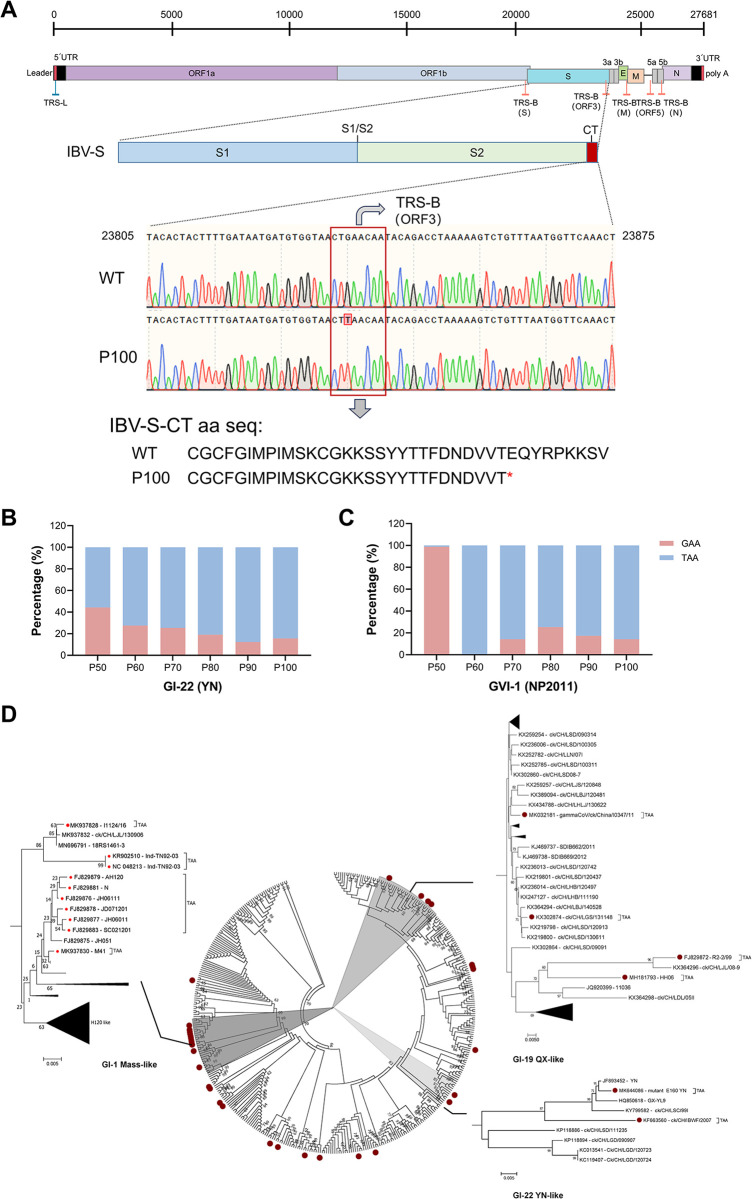
The adaptive mutation in the S gene arises during the embryo attenuation process. (A) Detailed depiction of mutation sites within viral genomes. (B, C) Assessment of the mutation frequency in the IBV YN (B) and NP2011 (C) strains propagated in chicken embryos. The strains serially passaged in ECEs (P50–100) were sequenced every 10 passages, and the percentage of the single nucleotide variation (%) was quantified using next-generation sequencing. (D) Phylogenetic analysis of the S gene of 401 IBV strains using data from NCBI. Mutants possessing with GAA to TAA mutation are marked with red dots.

We began by characterizing the genomic feature of the chicken-embryo-attenuated IBV strains YN and NP2011 (S1 and S2 Tables). We found that these IBV isolates shared a mutation at the 3’ end of the S gene, which involved a single nucleotide change from GAA, encoding glutamic acid, to TAA, encoding a stop codon (Fig 1A). We found that the incidence of this mutation increased with passage number, indicating the dominance of this mutant strain in the ECEs (Fig 1B and 1C). We next performed sequence alignment and phylogenetic tree construction using an NCBI dataset comprising 401 different genotype IBV strains and found that this mutation was highly prevalent in IBV strains (Fig 1D). These observations indicate that the identified mutation is not a random genetic alteration but rather an advantageous adaptation, which occurs when IBV is placed under natural selection pressure within the chicken embryo environment.

### The adaptive mutation decreases virulence in chickens and increases IBV adaptation in ECEs

**Fig 2:**
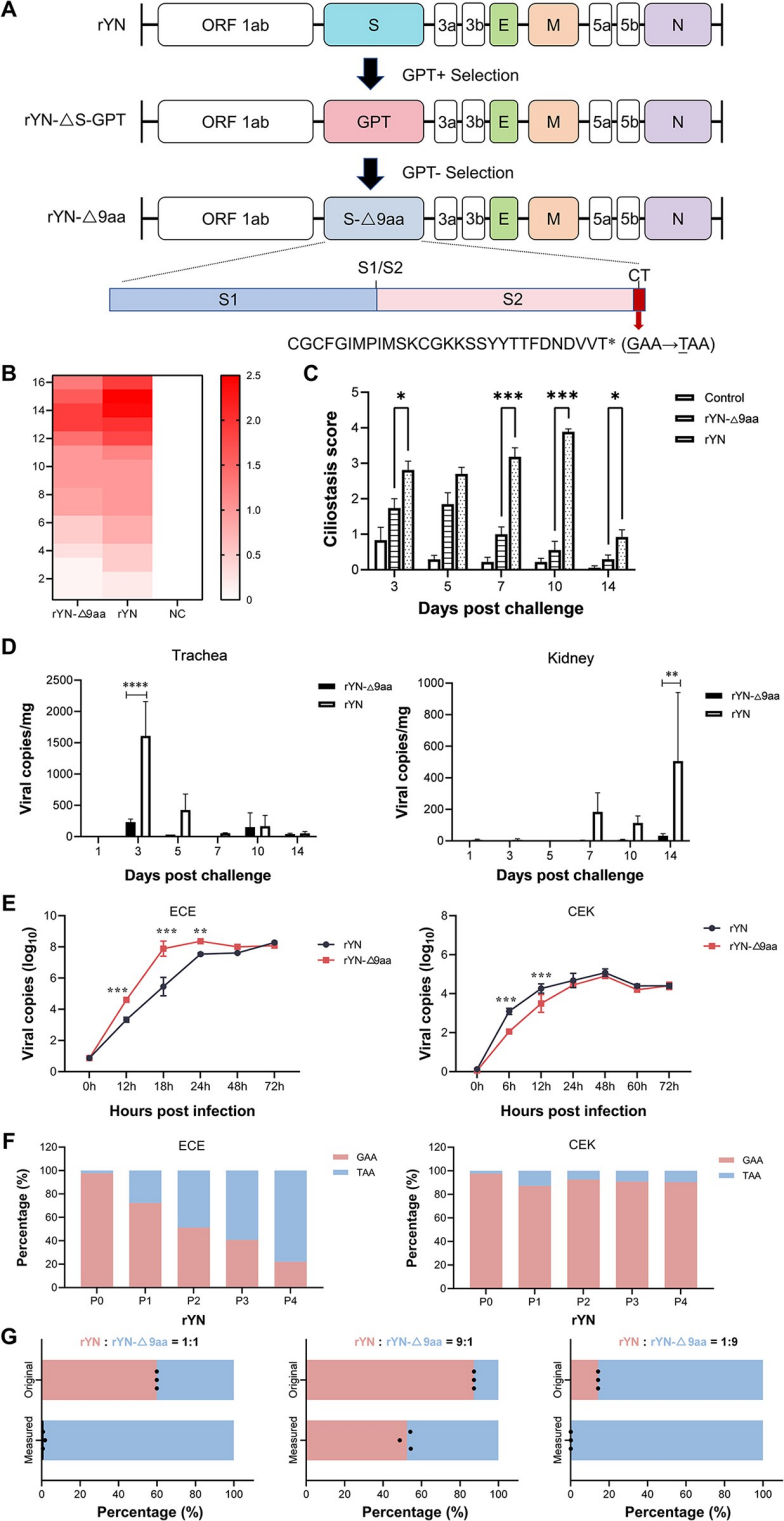
The pathogenicity of recombinant IBVs in SPF chickens and their replication in CEK cells and ECEs. (A) Schematic diagram of the rYN-Δ9aa rescue experiments and the location of mutations in the viral genomes. (B) A heatmap representation of the clinical symptoms of SPF chickens infected with rYN-WT, rYN-Δ9aa, and PBS. The daily clinical sign scores were recorded. The following scoring system was employed: 0 (normal), 1 (slight nasal discharge, mild shaking, and minor lacrimation), 2 (watery feces, depressive behavior, coughing or sneezing), 3 (severe nasal discharge, pronounced depression, mouth breathing, or tracheal rales), and 4 (death). (C) Tracheal ciliostasis evaluation. Ciliary activity in the trachea was assessed and scored at 3, 5, 7, 10 and 14 dpc. Scoring: 0 (full ciliary movement in the entire tracheal section), 1 (75% - 100% of normal ciliary movement), 2 (50% - 75% of normal ciliary movement), 3 (25% - 50% of normal ciliary movement), and 4 (< 25% of normal ciliary movement). The average ciliostasis scores were calculated for each group. (D) Viral load quantification post-challenge. RT-qPCR was used to detect viral RNA in the tracheal and kidney tissues harvested from each group of chickens. (E) Kinetic growth profiles of rYN-WT and rYN-Δ9aa in ECEs and CEK cells. Allantoic fluid or cell culture supernatants were collected at specified timepoints for viral load estimation via RT-qPCR. (F) Assessment of the GAA to TAA mutation frequency in the rYN-WT strain propagated in ECEs or CEK cells. The rYN-WT strain was serially passaged in ECEs or CEK cells five times. The S gene was sequenced after each passage and the extent of single nucleotide variation (%) was quantified using next-generation sequencing. (G) Results of the growth competition assay between rYN-WT (pink) and rYN-Δ9aa (blue) when they were combined at 1:1 (left), 9:1 (middle), and 1:9 (right). The percentage was calculated based on next-generation sequencing data.

We subsequently used our previously established reverse genetic system based on the recombinant IBV YN wildtype strain (rYN-WT) to construct and rescue the recombinant IBV strain rYN-Δ9aa by replacing the GAA in the 3’ end of the S gene with TAA (Fig 2A). We analyzed and compared the pathogenicities of the rYN-WT and rYN-Δ9aa strains in 1-week-old SPF chicks in terms of clinical symptoms, tracheal ciliostasis, and viral tissue distribution (Fig 2B–2D). The clinical symptom score of the rYN-Δ9aa-infected group was considerably lower than that of the rYN-WT-challenged group during the 14-day observation period (Fig 2B). The parental rYN-WT strain caused more extensive and severe damage to the host compared to rYN-Δ9aa strain, which only caused limited damage to the tracheal cilia (Fig 2C). The viral load of the trachea and kidney examined was also markedly lower in the rYN-Δ9aa group than in the rYN-WT group (Fig 2D). The results indicate that the G to T mutation in the S gene significantly reduces the pathogenicity of IBV in SPF chickens.

Next, the growth characteristics of recombinant strains were compared in both ECEs and chicken embryo kidney (CEK) cells using a multistep growth curve. In ECEs, the growth kinetics of the rYN-Δ9aa strain were markedly more rapid than those of the rYN-WT strain between infection initiation and 18 hours post-infection (hpi). Moreover, rYN-Δ9aa reached its growth peak within 24 hpi. Conversely, in CEK cells, the growth rate of rYN-Δ9aa was significantly lower than that of rYN-WT between infection initiation and 12 hpi (Fig 2E). We subsequently observed a unique phenomenon during the viral rescue process: a rapid GAA to TAA conversion occurred when the original rescued virus rYN-WT (at passage P0) was passaged in ECEs, resulting in a mixture of GAA- and TAA-containing quasi-species. We found that the proportion of TAA mutant viruses increased rapidly from 2% (at P0) to 77.96% (at P4). By contrast, the proportion of TAA mutant viruses in CEK cells remained below 10% after five passages (Fig 2F). To further compare the adaptability of the rYN-Δ9aa and rYN-WT strains in ECEs, a multiple-cycle growth competition assay was conducted and next-generation sequencing was used to determine the relative proportions of each virus. The results showed that the proportion rYN-Δ9aa virions increased markedly after one passage in ECEs (Fig 2G). Moreover, the proportion of YN-Δ9aa virions in ECEs was consistently significantly higher, irrespective of whether the starting rYN-WT to rYN-Δ9aa ratio was 1:1 or 1:9. Collectively, these data suggest that the adaptive mutation reduced the pathogenicity of IBV in SPF chickens and increased its adaptability in ECEs.

### The KKSV motif is crucial for viral replication in CEK cells but not for viral adaptability in ECEs

**Fig 3:**
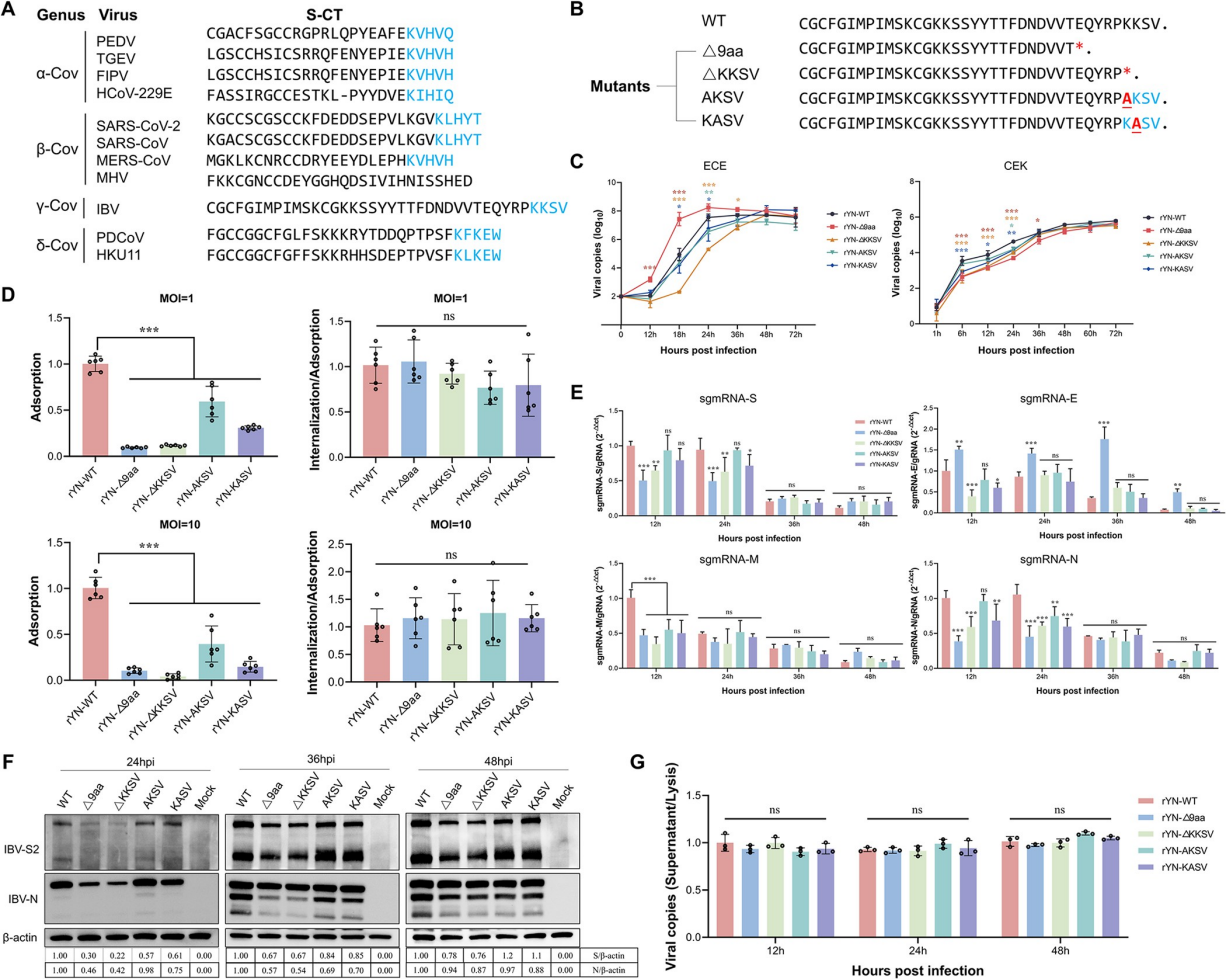
Deletion of the 9-aa sequence or the KKSV motif impairs early IBV replication in CEK cells by reducing viral invasion efficiency. (A) Comparative analysis of the cytoplasmic tails of S proteins across various coronaviruses. The ERRS motifs are highlighted in blue. The viruses compared included the porcine epidemic diarrhea virus (PEDV), transmissible gastroenteritis virus (TGEV), feline infectious peritonitis virus (FIPV), human CoV (HCoV), severe acute respiratory syndrome CoV-2 (SARS-CoV-2), severe acute respiratory syndrome CoV (SARS-CoV), middle east respiratory syndrome CoV (MERS-CoV), murine hepatitis virus (MHV), porcine deltacoronavirus (PDCoV), and bulbul CoV (HKU11). (B) Diagrammatic representation of mutations in the S protein sequences. Mutated residues are indicated in red, with asterisks (*) denoting termination mutations. (C) Growth kinetics of recombinant IBVs in ECEs and CEK cells. Allantoic fluid or cell culture supernatants were collected at specified timepoints for viral load estimation via RT-qPCR. (D) The adsorption and internalization efficiency of mutant viruses. CEK cells were infected with each virus at an MOI of 1 or 10; adsorption levels were assessed as described in the relevant materials and methods section. Internalization efficiency was calculated as the ratio of internalized to adsorbed virus. (E) Analysis of viral sgmRNA synthesis. CEK cells infected with recombinant IBVs were harvested for viral RNA extraction. The amounts of sgmRNA species encoding S, E, M, and N were quantified using RT-qPCR and expressed as a ratio relative to the amount of gRNA. (F) Protein synthesis in recombinant IBV-infected CEK cells. Cells infected with recombinant IBVs at an MOI of 0.01 were collected at specified post-infection timepoints for western blotting analysis, using β-actin as a loading control. (G) Release efficiency of mutant viruses. CEK cells infected with recombinant IBVs at an MOI of 0.01 were collected at specific timepoints. The viral copies in the supernatant and lysed cells were quantified by RT-qPCR, with the extent of viral release defined as the ratio of viral copies in the supernatant to those in the cell lysate. *P*-values were calculated using the one-way ANOVA; ns, not significant; *, *P* < 0.05; **, *P* < 0.01; ***, *P* < 0.001. All experiments were performed in triplicate.

As previously mentioned, the G to T mutation resulted in a 9-aa deletion in the CT of the IBV S protein. This 9-aa sequence contained a CoV-conserved dilysine trafficking motif, KKSV, which plays an important role in intracellular protein trafficking (Fig 3A). We therefore wondered whether the attenuated viral replication in CEK cells and the reduced virulence in SPF chickens caused by the 9-aa deletion were related to the deletion of the KKSV motif. To this end, we generated recombinant viruses carrying various mutations within the KKSV motif (rYN-ΔKKSV, rYN-AKSV, and rYN-KASV) and then rescued them using the aforementioned rescue strategy (Fig 3B). The multi-step growth kinetics of the viruses were assessed in CEK cells and ECEs (Fig 3C). At 6–24 hpi, the rYN-Δ9aa strain and KKSV motif mutants (particularly rYN-ΔKKSV variant) exhibited significantly weaker growth in CEK cells than the rYN-WT virus. Meanwhile, a variety of growth patterns were observed in ECEs between initiation of infection and 24 hpi, with rYN-Δ9aa exhibiting the fastest growth, and the KKSV mutants the slowest growth. These findings suggest that the KKSV motif is crucial for IBV replication within CEK cells but not for embryo adaptability.

### Deletion of the 9-aa sequence or the KKSV motif reduces IBV invasion efficiency in CEK cells

To determine which step of the viral replication cycle was affected by the G to T mutation or KKSV deletion, we examined each stage individually. We began by studying viral adsorption and internalization, which constitute the initial steps of the viral replication cycle, by incubating the virus with CEK cells at 4°C for 1 hour at a multiplicity of infection (MOI) of 1. At this low temperature, the virus can bind to the host cell but is not internalized. Once infected, the cells were immediately washed with chilled phosphate-buffered saline (PBS) to remove unbound virus. The viral RNA level in the cell lysates was detected by quantitative (q)PCR, and normalized against the expression of the GAPDH housekeeping gene. The results showed that the deletion or mutation of the 9-aa sequence or KKSV motif in the CT of the S protein significantly decreased the adsorption capacity of IBV. To exclude the possibility that the viral dose affected adsorption, we repeated the viral adsorption assays at an MOI of 10. The results confirmed that the reduction in IBV adsorption capacity was independent of the infection dose. We next performed the viral internalization assay. Briefly, the CEK cells were incubated with the virus at 4°C for 1 hour, washed with PBS, and then incubated at 37°C for 1 hour to allow the bound viruses to be internalized. The cells were washed again with PBS (to remove any cell-surface-bound virus), before being lysed and subjected to viral detection by qPCR. The results showed that there was no significant difference in the internalization abilities of the mutants and rYN-WT strains (Fig 3D).

We next examined the levels of subgenomic (sgm)RNA via real-time (RT)-qPCR using specific primers targeting the IBV structural protein genes. Differences in the expression levels of sgmRNA-S, -M, and -N were mainly observed at 12 and 24 hpi, with the rYN-Δ9aa group exhibiting the lowest levels of expression, followed by rYN-ΔKKSV, rYN-KASV, and rYN-AKSV. At 12 hpi, the sgmRNA-E level of rYN-Δ9aa was particularly high compared with that of rYN-WT, while the sgmRNA-E levels of rYN-ΔKKSV and rYN-KASV were lower than that of rYN-WT at this timepoint. Given that the 9-aa deletion lies in the core sequence of the ORF3 TRS-B, we hypothesized that it might increase the efficiency of the discontinuous transcription process, yielding higher levels of sgmRNA-E transcripts during infection. However, no significant differences in sgmRNA-E were seen between rYN-ΔKKSV, rYN-AKSV, rYN-KASV, and rYN-WT at 36 and 48 hpi (Fig 3E). Western blotting analysis confirmed the RT-qPCR results by showed that the protein levels corresponded to the sgmRNA levels. In the rYN-WT group, the expression of the S and N proteins was detectable around 24 hpi. Meanwhile, the rYN-Δ9aa, rYN-ΔKKSV, rYN-AKSV, and rYN-KASV groups all expressed lower levels of S and N proteins than the rYN-WT group (Fig 3F). We next evaluated differences in the extent of viral release by quantifying the ratio of IBV copies in the cell culture supernatant to that in the cell lysate. We observed no significant differences in the capacity of the WT and mutant IBV strains to release infectious viral particles into the supernatant at the different timepoints (Fig 3G). Collectively, these results demonstrate that the deletion of the 9-aa sequence or the KKSV motif mainly affect viral invasion efficiency, meaning that viral replication was impaired in CEK cells in the early stages of infection, without greatly impacting virion release at later stages of the infection process.

### KKSV motif deletion in the CT domain inhibits S protein localization to the ERGIC and increases cell surface membrane expression

**Fig 4:**
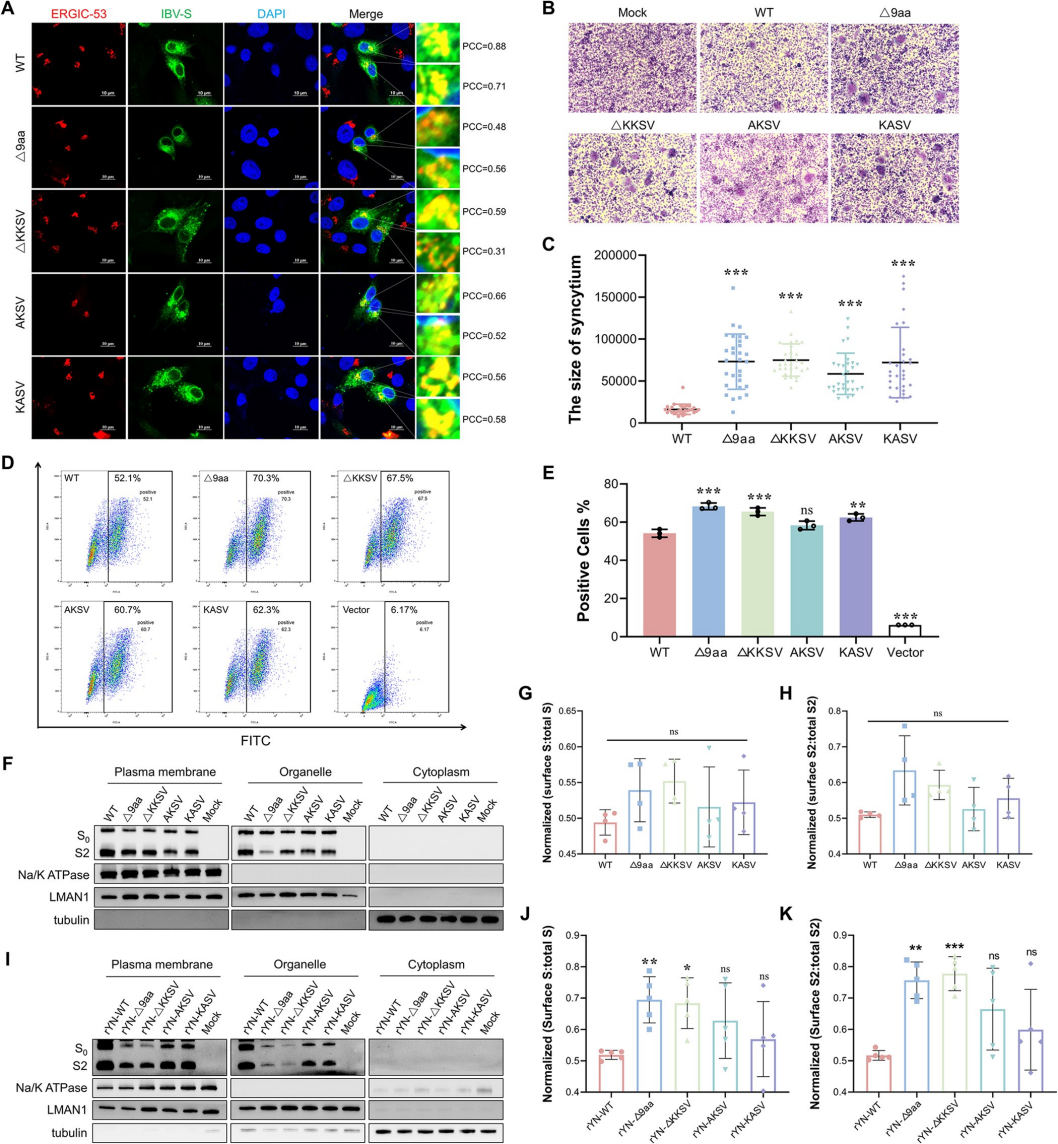
Deletion of the 9-aa sequence and the KKSV motif increases IBV S protein expression on the cell surface. (A) Examination of the subcellular localization of the S protein and its mutants in the ERGIC. BHK-21 cells transfected with the indicated plasmids were fixed at 24 hours post-transfection for IFA. The extent of colocalization of S proteins with the ERGIC was quantified using PCC. Scale bar: 10 μm. (B) Induction of syncytia by the IBV S protein and its mutants. BHK-21 cells were transfected with the indicated plasmids, fixed with methanol at 36 hours post-transfection, and subjected to Giemsa staining. Syncytium size was normalized and quantified using ImageJ software. (C) Measurement of syncytia sizes based on 30 individual samples per variant. (D) Flow cytometric evaluation of the cell surface expression of S proteins. HEK-293T cells were collected at 36 hours post-transfection, and labeled with an anti-Flag antibody and an Alexa Fluor 488 (AF488)-conjugated goat anti-rabbit IgG, without cell permeabilization. Cell surface S protein expression was then quantified (as the percentage of AF488^+^ cells) using a BD FACSCanto II flow cytometer and FlowJo software. (E) Quantitative assessment of cell surface S protein expression; the values shown were derived from three independent experiments. *P* values were determined using the one-way ANOVA, with significance levels indicated as *, *P* < 0.05; **, *P* < 0.01; ***, *P* < 0.001. (F) Western blotting analysis of cell surface S protein expression. HEK-293T cells were harvested at 36 hours post-transfection, and the proteins from various cellular fractions were isolated. The Na/K ATPase was used as a plasma membrane loading control, LMAN1 was used as a total membrane marker, and tubulin was used as a cytoplasmic loading control. (G) Quantification of S protein expression in the cell surface. S protein levels were calculated as the sum of the amounts of full-length S0 and cleaved S2 products, whereby the amount of plasma membrane S indicated cell surface expression and total S reflected the total amount of S protein across all cellular fractions. (H) Quantitative assessment of S2 protein expression on the cell surface using grayscale scanning. (I) The cell surface expression of the S protein during IBV infection was determined by western blotting. CEK cells were infected with IBV at an MOI of 0.01. At 36 hours post-infection, the proteins from the different cellular fractions were isolated. (J) Quantitative analysis of cell surface S protein expression during IBV infection. (K) Quantitative analysis of cell surface S2 protein expression during IBV infection. *P*-values were calculated using the one-way ANOVA and denoted as ns (not significant), *, *P* < 0.05; **, *P* < 0.01; ***, *P* < 0.001.

To elucidate the impact of the KKSV motif deletion on the intracellular sorting of the S protein, we constructed five plasmids (pRK5-Flag-WT, pRK5-Flag-Δ9aa, pRK5-Flag-ΔKKSV, pRK5-Flag-AKSV, and pRK5-Flag-KASV), which either bore a Flag-tagged WT S gene (YN) or an S gene with one of four CT-targeting mutations. We initially used these plasmids to investigate the role of the KKSV motif in ERGIC retention. To this end, BHK-21 cells were transiently transfected with each of the plasmids, fixed at 24 hpi, and then stained with antibodies against Flag and the ERGIC marker ERGIC-53 (Fig 4A). Pearson’s correlation coefficient (PCC) was employed to quantify the degree of colocalization between the Flag-S protein and the ERGIC signal, with higher PCC values indicating increased colocalization. Our results demonstrated that the PCC values for the S protein with the intact KKSV motif (S-WT) were significantly higher than those of the other S protein mutants (Δ9aa, ΔKKSV, AKSV, and KASV). This result indicates that while the S-WT was able to effectively localize to the ERGIC, the mutants exhibited colocalization defects.

We subsequently quantified S protein levels on the surface of cells transfected with each of the pRK5-Flag plasmids, using a combination of syncytium induction, flow cytometry, and plasma membrane protein extraction assays. The syncytium induction assay was performed to assess the extent of cell fusion induced by the proteolytic cleavage of the surface S protein. The findings demonstrated that all the cells expressing S proteins with CT mutations exhibited notably larger syncytia than those expressing the WT S protein (Fig 4B and 4C). Flow cytometry was then used to monitor the transient expression of S protein on the cell membrane of HEK-293T cells. The results revealed that the Δ9aa and KKSV motif deletion groups had higher levels of surface S protein expression than the WT group (Fig 4D and 4E). The cell membrane, organelles, and cytoplasm were subsequently separated from cell lysates and analyzed by western blotting to assess the S protein levels of each compartment (Fig 4F); the ratio of the amount of S protein within each compartment to total amount of S protein was quantified. The results of the western blotting evaluation were consistent with those of the flow cytometric analysis. They showed that the Δ9aa and ΔKKSV groups had higher levels of S protein (particularly for S2) on the cell membrane than in the membranes of organelles (Fig 4G and 4H). Conversely, the WT group and the single point mutation groups (AKSV, KASV) demonstrated similar levels of S protein expression in both the cell and organelle membranes. These trends were consistently observed in independently replicated experiments. At the viral level, these observations were supported by similar experiment (Fig 4I). For instance, the rYN-Δ9aa and rYN-ΔKKSV groups had a significantly higher proportion of S protein on the cell membrane surface (again particularly for S2) than the WT group (Fig 4J and 4K). Viruses with a single K mutation also displayed increased cell surface S protein expression levels; however, the differences in relation to WT were not significant. Taken together, these findings underscore the pivotal role of the KKSV motif in regulating IBV S protein expression on the cell membrane surface.

### Deletion of the 9-aa sequence impairs S protein incorporation into virions

**Fig 5:**
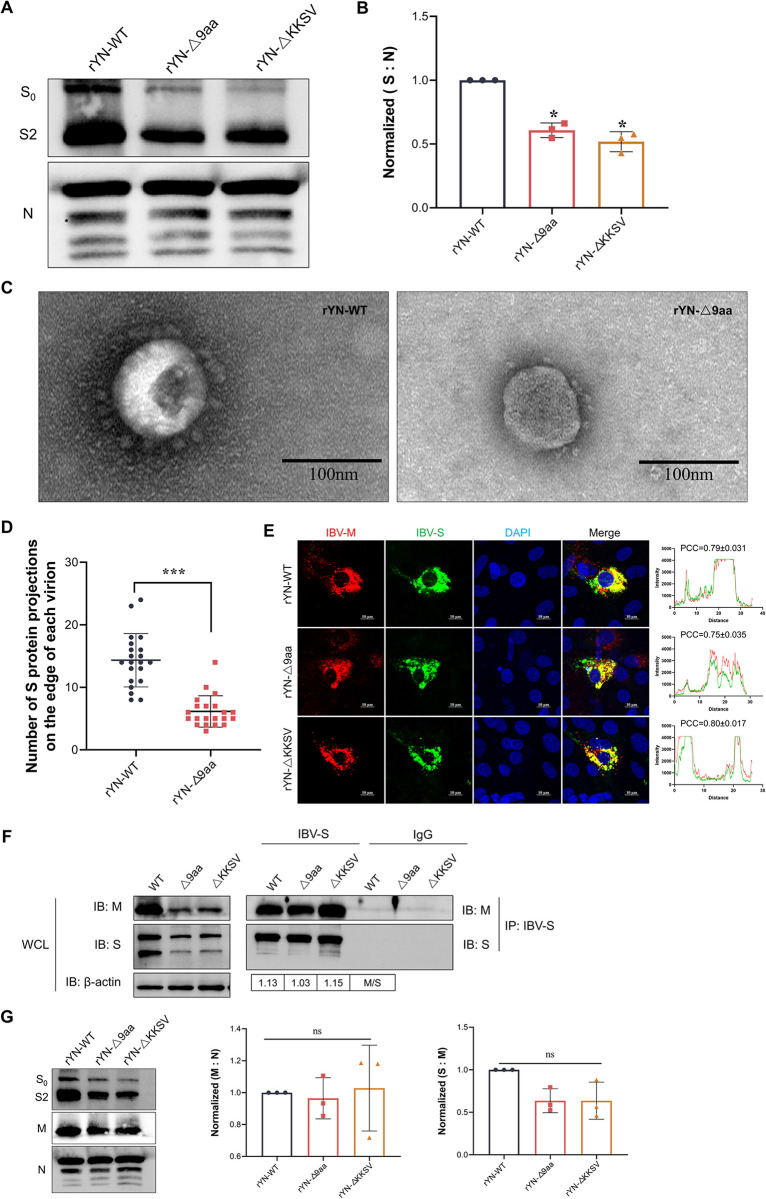
Deletion of the 9-aa sequence and the KKSV motif impacts viral assembly. (A) Western blotting analysis of S protein incorporation into viral particles. Virions were concentrated and purified using sucrose gradient centrifugation. S protein expression in IBV particles was then detected by western blotting, with the viral N protein serving as the loading control. (B) Grayscale scanning quantification of the ratio of S to N from the western blotting data shown in (A). (C) TEM images of purified rYN-WT and rYN-Δ9aa virions. Scale bar: 100 nm. (D) Quantification of S projections on the surface of individual virions. A total of 20 particles displaying S projections were randomly selected for counting from the TEM images. (E) IFA staining for S and M protein colocalization in CEK cells during infection with recombinant IBVs. Cells infected at an MOI of 0.01 were fixed at 36 hours post-infection and stained with antibodies against S (green) and M (red) proteins. Scale bar: 10 μm. PCC was used to quantify S and M protein colocalization. (F) Analysis of S and M protein interactions. CEK cells were infected with recombinant IBVs at an MOI of 0.01. The cell lysates were immunoprecipitated with anti-S antibodies, and then the levels of S and M protein were detected by western blotting. (G) Western blotting analysis of M protein expression in viral particles. Virions were concentrated and purified using sucrose gradient centrifugation. S, M, and N protein expression in viral particles was detected by western blotting, with viral N protein serving as a loading control. Statistical analysis was performed using the one-way ANOVA (n = 3) and significance was denoted as ns (not significant), *, *P* < 0.05; **, *P* < 0.01; ***, *P* < 0.001. All experiments were conducted in triplicate.

To assess the influence of enhanced translocation of S proteins to the cellular membrane surface on their incorporation into viral particles, we next quantified S proteins on purified viral particles using western blotting. To mitigate any potential inconsistencies in sample preparation, we cultivated and purified the three recombinant IBVs (rYN-WT, rYN-Δ9aa, and rYN-ΔKKSV) simultaneously. The IBV N protein was used as an internal standard to ensure that S protein expression was assessed in equal quantities of viral particles. We found that the rYN-Δ9aa and rYN-ΔKKSV variants integrated considerably less S protein into virions than rYN-WT (Fig 5A and 5B). Transmission electron microscopy (TEM) was used to visualize the presence of S protein projections on the surface of individual virions. The TEM results showed that rYN-Δ9aa virions had fewer S protein projections than the virions in rYN-WT (Fig 5C). Moreover, the rYN-Δ9aa had significantly fewer S projections per virion than the rYN-WT (Fig 5D). These findings indicate that the deletion of the 9-aa or KKSV motifs from the S protein impairs its incorporation into IBV virions, leading to defective viral production.

Additionally, we wanted to determine the potential impact of the 9-aa/KKSV deletion on the interaction between the S and M proteins, as well as their incorporation into viral particles. Confocal microscopy and immunoprecipitation revealed no significant differences in the colocalization and interaction of the two proteins among the rYN-WT, rYN-Δ9aa, and rYN-ΔKKSV (Fig 5E and 5F). Moreover, the rate of M assembly and the S to M ratio remained unchanged among the experimental groups (Fig 5G). These findings suggest that mutations in the CT of the S protein do not influence the interaction between the S and M proteins, nor their assembly into viral particles.

### Deletion of the 9-aa sequence or KKSV motif reduces IBV virulence in SPF chickens

**Fig 6:**
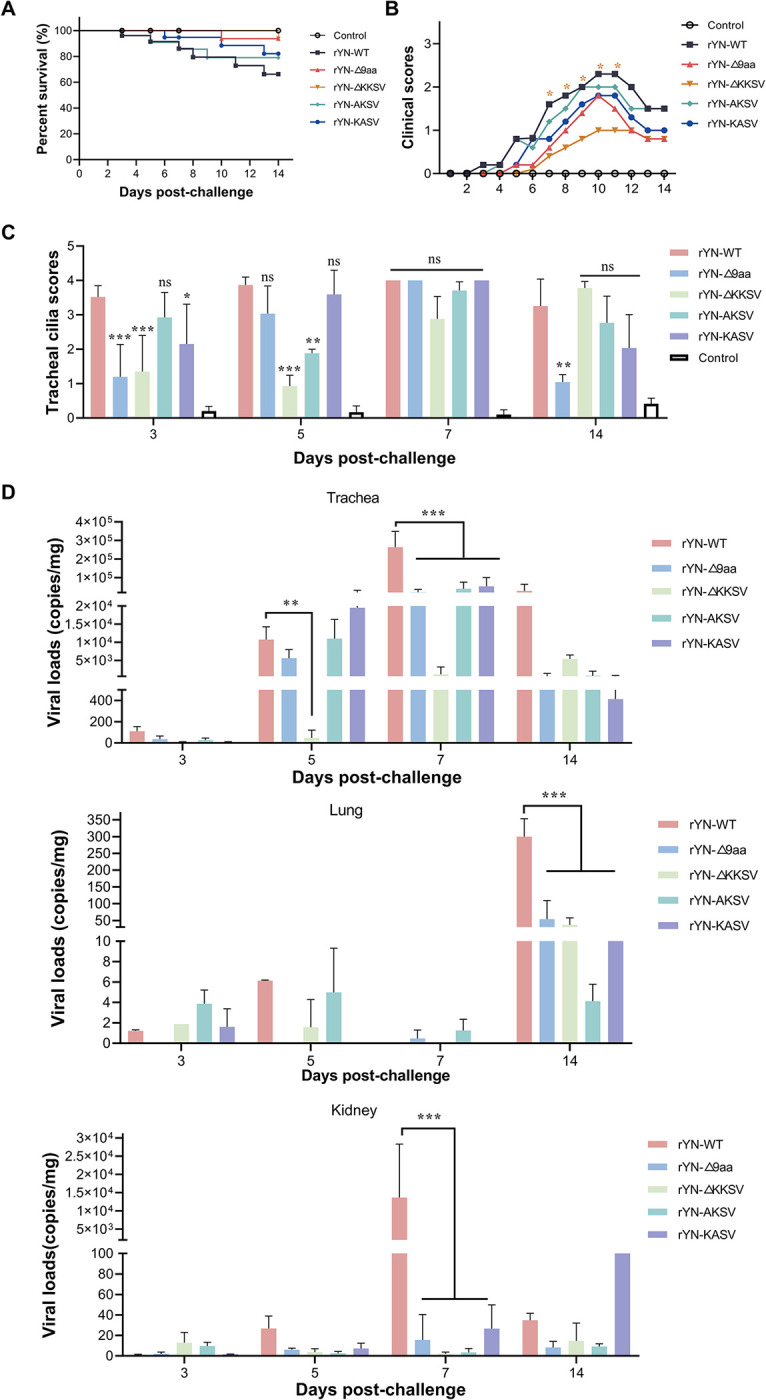
Deletion of the 9-aa sequence and the KKSV motif reduces the virulence of recombinant IBVs. (A) Survival curve depicting the percentage survival in each group over a 14-day observation period. (B) Daily clinical sign scores. Scoring system: 0 (normal), 1 (slight nasal discharge, mild shaking, and minor lacrimation), 2 (watery feces, depressive behavior, coughing or sneezing), 3 (severe nasal discharge, pronounced depression, mouth breathing, or tracheal rales), and 4 (death). (C) Tracheal ciliostasis evaluation. Ciliary activity in the trachea was assessed and scored at 3, 5, 7, and 14 dpc. Scoring: 0 (full ciliary movement in the entire tracheal section), 1 (75% - 100% of normal ciliary movement), 2 (50% - 75% of normal ciliary movement), 3 (25% - 50% of normal ciliary movement), and 4 (< 25% of normal ciliary movement). The average ciliostasis scores were calculated for each group. (D) Viral load quantification post-challenge. RT-qPCR was used to detect viral RNA in the tracheal, lung, and kidney tissues collected from each group.

**Fig 7:**
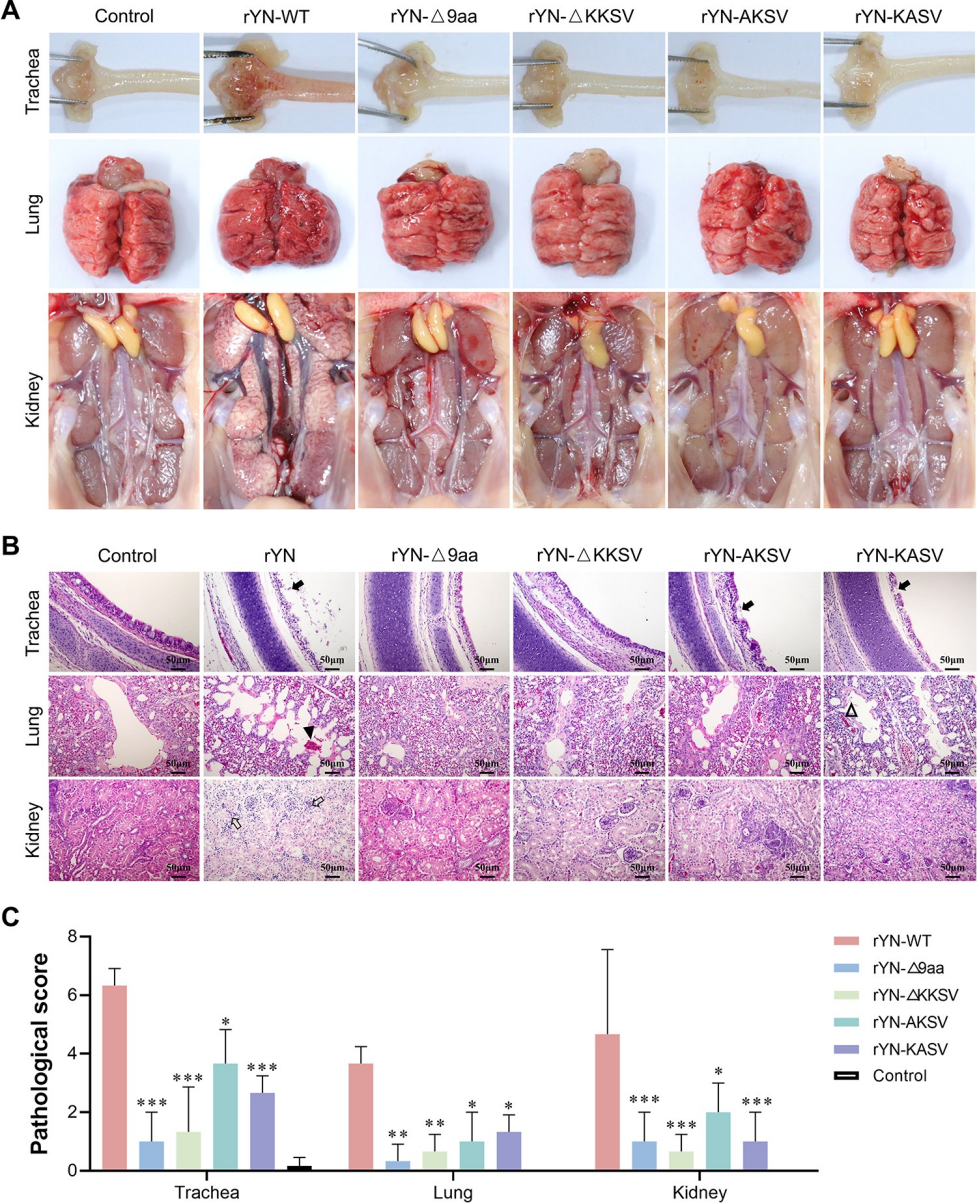
Examination of tissue lesions and histopathologic changes in SPF chickens challenged with recombinant IBVs. (A) Examination of lesions in tracheal, lung, and kidney tissues at 7 days post-infection. (B) Detailed histopathological analysis results; notable features included extensive dropout, degeneration, and necrosis of ciliated epithelial cells (indicated by black arrows), hemorrhage in the bronchial lumen (marked by black triangles), exfoliated fibroblasts and mucus in the bronchial lumen (denoted by open triangles), and renal tubulointerstitial lymphocyte infiltration (shown with open arrows). Scale bar: 50 μm. (C) The microscopic pathological changes were scored as follows: 0 (no microscopic lesions), 1–3 (mild lesions), 4–6 (moderate lesions), and 7–10 (severe and extensive lesions).

Lastly, we comprehensively analyzed the impact of the 9-aa/KKSV deletion on viral pathogenicity. We compared the mortality rates and clinical symptom scores of 1-day-old SPF chicks infected with different recombinant viruses over a 14-day observation period (Fig 6A and 6B). The chickens infected with rYN-WT and rYN-AKSV displayed severe clinical symptoms, resulting in mortality rates of 33.81% and 21.02%, respectively. The rYN-KASV group showed lower clinical scores and a mortality rate of 17.89%. By contrast, the groups infected with rYN-Δ9aa and rYN-ΔKKSV exhibited only mild clinical symptoms and both had a mortality rate of 6.25%. Tracheal ciliostasis analysis revealed that the rYN-WT group had the most severe tracheal damage on various days post-challenge (dpc), as evidenced by the highest ciliary injury score, while the rYN-Δ9aa and rYN-ΔKKSV groups had lower ciliary injury scores (Fig 6C). RT-qPCR analysis of viral distribution in tissues indicated that the tracheal, lung, and kidney tissues (the key organs targeted by IBV) of SPF chickens in the rYN-Δ9aa and KKSV motif mutation groups had lower viral loads that the same tissues of chickens in the rYN-WT group. The most significant reduction in viral load was observed in the trachea at 5 and 7 dpc in the rYN-ΔKKSV group (Fig 6D). Significant tissue damage (e.g., pronounced tracheal and lung hemorrhages, along with urate deposition in the kidneys) was observed in the trachea, lung, and kidneys of the rYN-WT group (Fig 7A). By contrast, minimal tissue damage was observed in the rYN-Δ9aa and KKSV motif mutation groups. In accordance, the histopathology results showed notable exfoliation of the ciliated epithelium in the trachea, hemorrhage and mucus exudation in the bronchial cavity of the lung, as well as a large number of infiltrating inflammatory cells in the kidney in rYN-WT (Fig 7B and 7C). Collectively, these findings suggest that the recombinant IBV lacking the 9-aa or KKSV motifs exhibits reduced virulence in SPF chickens.

### The adaptive G to T mutation converts the TRS-B of ORF3 to TRS-L and promotes ORF3 transcription

**Fig 8:**
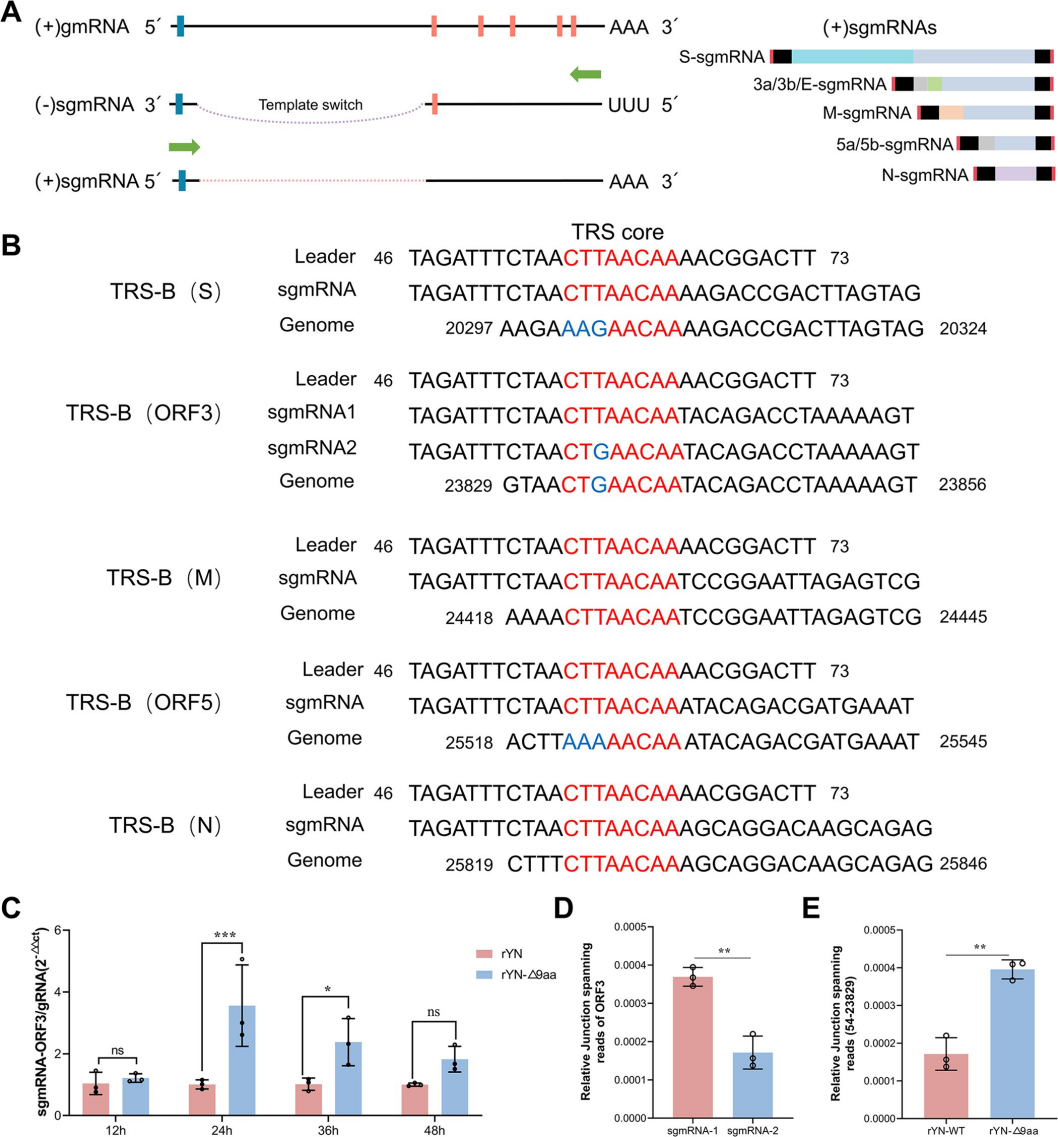
The adaptive G to T mutation promotes ORF3 transcription. (A) Schematic illustration of the IBV sgmRNA synthesis method. (B) Alignment of TRS-L, sgmRNAs, and TRS-B to illustrate the template switching pattern in IBV. The sgmRNAs were amplified by RT-PCR using primers specific to the leader sequence and the respective sgmRNAs. Red letters indicate the canonical CS of TRS-B and TRS-L; and blue letters denote the non-canonical CS of TRS-B. (C) Analysis of viral ORF3-sgmRNA synthesis. CEK cells infected with recombinant IBVs were harvested for viral RNA extraction. The ratios of ORF3 sgmRNAs relative to gRNA were quantified using RT-qPCR. (D) The ratio of the two ORF3 transcripts in the YN strain. The expression levels of each sgmRNA were normalized against the total number of viral-genome-mapped reads at each read junction. (E) Relative junction-spanning reads at 54–23,829.

We decided to investigate the mechanisms underlying the abnormal increase in the level of sgmRNA-E in the mutant IBV strain in Fig 3E. During the transcription process, RdRp initiates RNA synthesis on the negative strand. Upon encountering the TRS-B, which is positioned upstream of each gene, RdRp orchestrates the template switch to the TRS-L (Fig 8A). Given that the TRS-B acts as a cue for RdRp to switch templates, recombination events are more likely to occur at or near these TRS-B sites. ORF3 encompasses the 3a, 3b, and E genes and has a TRS-B located at the 3’ end of the S gene. Further investigation revealed that the G to T mutation site was also situated within the TRS-B of ORF3. Specifically, the mutation changed the ORF3 TRS-B from CTGAACAA to CTTAACAA, making it almost identical to the TRS-L.

We then explored the impact of this TRS-B to -L transformation on ORF3 function. First, we analyzed the sgmRNA products in IBV using leader-body junction RT-PCR. Using next-generation sequencing and sequence alignment tools, we discovered the presence of two ORF3 subgenomic transcripts in the rYN-WT strain (Fig 8B). During discontinuous transcription, template switching does not always occur at the same location, especially when the pairing between TRS-B and TRS-L is incomplete. To investigate whether different switching sites in ORF3 led to the generation of two transcripts, we further analyzed the template switching sites using RNA-sequencing (RNA-seq). We found that the rYN-WT strain has two jumping sites in ORF3, at positions 23,829 and 23,835 (Table 1). However, in the rYN-Δ9aa strain, template switching did not occur after the site of the G to T mutation at position 23,835; this resulted in the transcription of only one ORF3 subgenomic transcript (Table 2). Complementary pairing between TRS-B and TRS-L is crucial for efficient of subgenomic transcription during the synthesis of CoV sgmRNAs. Thus, we next examined the subgenomic transcription level of ORF3 using RT-qPCR. Consistent with the results of IBV E gene expression, the subgenomic level of ORF3 were significantly higher in the rYN-Δ9aa strain (Fig 8C). We next compared the proportions of the two ORF3 subgenomic transcripts in rYN-WT using RNA-seq and found that the transcriptional efficiency of CTTAACAA was significantly higher than that of CTGAACAA (Fig 8D). Since the rYN-Δ9aa mutant contained only one switching site at position 23,829, we compared the transcriptional efficiency of this switching site between the two strains (Fig 8E). The results indicated that the transcriptional efficiency of the ORF3 in the rYN-9aa strain was significantly higher than that in rYN-WT. Additionally, we employed RNA-seq to compare the transcription levels of the S, M, N, and ORF5 genes between the rYN-WT and rYN-Δ9aa viral strains. Our analysis revealed no significant differences in the transcriptional profiles of these genes between the two strains (S1 Fig). Taken together, our research demonstrates that the G to T mutation at position 23,835 in IBV results in a perfect pairing between the TRS-B and TRS-L of ORF3, increasing the subgenomic transcription efficiency of the ORF3 gene.

**Table 1:** Canonical sgmRNAs of rYN.

sgmRNA name	Template switch junction		Junction length	TRS-core
	5´	3´		
S	60	20303	20244	CTTAACAA
ORF3-sgmRNA-1	60	23835	23776	CTTAACAA
ORF3-sgmRNA-2	54	23829	23776	CTGAACAA
M	55	24419	24365	CTTAACAA
ORF5	60	25524	25465	CTTAACAA
N	57	25822	25766	CTTAACAA

**Table 2:** Canonical sgmRNAs of rYN-Δ9aa.

sgmRNA name	Template switch junction		Junction length	TRS-core
	5´	3´		
S	60	20303	20244	CTTAACAA
ORF3-sgmRNA-1	54	23829	23776	CTTAACAA
M	55	24419	24365	CTTAACAA
ORF5	60	25524	25465	CTTAACAA
N	57	25822	25766	CTTAACAA

## Discussion

IBV is a highly transmissible CoV, which causes infectious bronchitis (IB) in chickens. Live attenuated IBV vaccines have been extensively employed to safeguard chickens against IB [20,21]. These vaccines are typically derived from the wild-type strain of IBV through successive passages in the chorioallantoic membranes of ECEs. The process of passaging IBV clinical isolates in ECEs often leads to the emergence of egg-adaptive mutations. It is generally believed that these mutations occur spontaneously during the process of attenuation in ECEs; moreover, the specific mutations vary among the different attenuated strains [31,32]. For instance, a comparative analysis of the genome sequences of virulent and attenuated variants of the avian IBV strain Ark DPI revealed 21 nucleotide differences, which resulted in 17 amino acid changes, primarily in the replicase 1a and S genes [33]. Another study investigated the complete genome sequences of three attenuated IBV strains (Ark, GA98, and Mass41) after they were passaged in ECEs, and found that 34.75%–43.66% of all mutations occurred in the nsp3 gene [34]. Our previous research also discovered an 82-nt deletion in the 5a gene during passaging, which was linked to viral attenuation [35]. In the present study, we identified a nucleotide (G to T) mutation at the 3’ end of the S gene of IBV during the serial passaging of the IBV isolates YN and NP2011 in chicken embryos. This mutation converted the glutamic acid (E) at position 1,159 of the S gene into a stop codon. Importantly, we also identified this mutation in other isolated IBV strains. A similar shift was reported in a QX-Type IBV strain Sczy3 after extensive passaging in CEK cells [36,37]. This observation implied that this mutation was not a random genetic event, but rather an adaptive mutation shaped by natural selection pressure.

Rescue experiment of the recombinant mutant strain rYN-Δ9aa showed that the adaptive mutation endowed IBV with a significant replication advantage in chicken embryos versus in CEK cells. Moreover, the high mutation rate of the virus yielded a heterogeneous population composed of numerous genetic variants [38]. Different environmental conditions exert distinct selective pressures, which shape viral adaptation to those specific environments. The S-Δ9aa mutation, which arose during serial passaging in ECEs, appears to be an adaptation to this specific setting; as such, it confers a replication advantage in ECEs. Similar adaptive mutations have been reported in other studies. For instance, the N501Y mutation in the S protein of SARS-CoV-2 increased viral adaptability in mice after six passages [39]. Moreover, a number of mutations were discovered in the murine norovirus (MNV) after six passages in HeLa cells, which were associated with enhanced adaptability the replication capacity [40]. However, adaptation to one environment can incur fitness costs that diminish adaptation to other environments [41]. For instance, MNV variants adapted to human cells showed reduced adaptability in murine BV2 cells and in mouse infections [40]. Similarly, a mutation in the capsid protein VP1-F106L of coxsackievirus B3 increased the viral replication rate in HeLa cells but decreased it in mice [42]. Thus, the adaptability of the IBV mutant to the ECE environment acts as a trade-off for the reduced adaptability in CEK cells and chicken hosts. This notion was confirmed by the results of the growth curve analysis in CEK cells and the pathogenicity tests in chickens. This study underscores the dynamic nature of viral evolution, where adaptive mutations can confer selective advantages in specific environments but may also compromise viral fitness in other contexts.

In the production of live attenuated vaccines for IBV, the attenuation of virulence is a critical step. Understanding the molecular basis of attenuation allows for the design of vaccines that are not only safe but also stable. In this study, we focused on elucidating how the adaptive G to T mutation in the CT region of the S protein contributes to the attenuation of IBV virulence. Numerous RNA viruses acquire truncation mutations in the CT of their glycoproteins during serial passaging as a common adaptive response to their environment. For example, an early termination codon was identified at the 3’ end of the S gene of the high-virulence PEDV strain PC22A after it was passaged 120 times in Vero cells; this mutation also caused a 9-aa truncation in the CT of the PEDV S protein [23]. Similarly, the recombinant vesicular stomatitis virus (rVSV) expressing the SADS-CoV S protein, acquired an 11-aa truncating deletion in the CT of the S protein after passaging in Huh7.5.1 cells [24]. Similar premature termination codons, which induced CT truncation, have been observed in the envelope glycoproteins of HIV-1, SIV, and EIAV [25,27,28]. These truncations generally decrease viral replication and pathogenicity in vivo. Thus, to further understand the impact of the G to T mutation on IBV virulence, we specifically examined the truncation of the IBV S protein CT. By investigating which specific factors contributed to the virus’s reduced replicative ability in CEK cells, we found that the deletion of either the 9-aa sequence or the KKSV motif significantly impaired the early phase of IBV replication in CEK cells. Characterization of the specific stages of the viral life cycle revealed that the deletion of the 9-aa sequence or the KKSV motif substantially diminished viral invasion efficiency, and thus, reduced the rates of early sgmRNA and protein synthesis in comparison with the WT strain.

Like many other viruses, CoVs rely on their surface glycoproteins to interact with host receptors and merge with cell membranes. Therefore, the S protein plays a crucial role in the invasion of coronaviruses [43–45]. Mechanistically, the KKSV motif is required for the retention of viral S proteins within the ERGIC, thereby restricting their transport to the cell surface. Consequently, the deletion or mutation of the KKSV motif promotes S protein accumulation at the cell membrane surface. A higher expression level of the surface subunit of a glycoprotein on the cell surface was found to enhance syncytium formation [46–48]. Syncytium induction experiments have shown that the levels of surface glycoprotein subunit expression on the cell surface correlate positively with syncytium formation. These findings were further supported by flow cytometric analyses, which detected S protein expression on the plasma membrane and the cytoplasm. However, it should be noted that not all S proteins can reach the cell membrane due to the presence of endocytosis motifs, which redirect a portion of the proteins back to the intracellular compartment. Previous research conducted on α-CoVs (e.g., TGEV and PEDV) and γ-CoVs (e.g., IBV) has demonstrated that the S proteins are primarily localized intracellularly and rarely appear on the cell surface when expressed independently [29,47,49]. By contrast, our findings revealed that 50% of the IBV S proteins were present on the cell membrane when cells were transfected with the plasmids encoding the IBV S protein alone. This discrepancy may arise from differences in the IBV S protein expression levels or the use of chimeric proteins containing solely the 11-aa sequence of the IBV S protein CT.

We found that altering S protein localization within the cell decreased its integration into viral particles, which consequently substantially reduced viral binding to the host cell receptor. In the present study, the amount of S proteins on recombinant IBV particles were measured using a combination of western blotting and TEM. The results revealed a notable decrease in the number of S proteins on the surface of viral particles harboring the adaptive mutation. One plausible explanation for this is that the absence of ERRS signal increases the translocation of S proteins to the cell surface, thereby reducing their access to the viral assembly site in the ERGIC. It is also important to consider the pivotal role played by M proteins in the recruitment of structural proteins during viral assembly. The interaction between the S and M proteins is vital for the successful incorporation of S proteins into virions [50–52]. Previous studies have shown that mutations in the ERRS sequence (KLHYT) of the SARS-CoV S protein can disrupt the S/M protein interaction and consequently impact S protein incorporation into viral particles [30,53]. To validate the notion that the G to T mutation reduced S protein incorporation into virions by disrupting the interaction between S and M proteins, we performed a series of experiments, including IFA and coimmunoprecipitation. Our results revealed that the deletion of the 9-aa sequence or the KKSV motif in the S protein CT did not significantly impact the S/M protein interaction during viral particle assembly. This finding aligns closely with that of another study, which also reported that the inactivation of the KKSV motif in the IBV S protein did not alter the S/M interaction [54]. Similarly, research on PEDV has demonstrated that the deletion of the ERRS motif in the S protein did not affect its interaction with the M protein [23]. Collectively, these findings suggest that although the deletion of the 9-aa sequence or the KKSV motif in the S protein CT may not alter the interaction between the S and M proteins, it still exerts a substantial effect on viral assembly.

We observed a significant reduction in the virulence of recombinant IBV strains, particularly rYN-Δ9aa and rYN-ΔKKSV, in comparison with the parental strain rYN-WT, in infection experiments with 1-day-old SPF chicks. This reduction in pathogenicity was also observed, albeit to a lesser extent, in infection experiments with the rYN-AKSV and rYN-KASV strains. These findings contradict those of Hou et al. [23], who found that the deletion of the ERRS motif (KVHVQ) had no impact on the pathogenicity of PEDV in piglets. This discrepancy may be attributed to the more classic nature of the KKxx form (found in the recombinant IBV strains used in the present study) versus the KxHxx form (found in PEDV) of the ERRS motif, which may decrease viral pathogenicity to a greater extent than its canonical counterpart. This hypothesis is substantiated by evidence that the S proteins of PEDV and SARS-CoV-2, which both have the KxHxx ERRS motif, exhibit a weaker affinity for COP I (a cellular protein that plays a crucial role in mediating the transport of cargo from the Golgi apparatus to the endoplasmic reticulum) than the S protein of IBV, which has the canonical KKxx motif [55,56]. Given these observations, we propose that the S-Δ9aa mutation could be used to effectively reduce the virulence of IBV strains and aid the development of IBV vaccines using reverse genetics methods.

The mutation from GAA to TAA within the S gene not only affects the cytoplasmic tail region of the S protein but also exerts an influence on the TRS-B of the ORF3, aligning TRS-B with the TRS-L. CoV TRSs are recognized as hotspots for mutations and recombination events [57]. Recent studies have identified TRS mutations in many of the emerging SARS-CoV-2 variants, which exhibit substantially reduced virulence [58]. Thus, the mutation in the ORF3 TRS-B could be another contributing factor to the attenuated pathogenicity of the IBV strain carrying the G to T mutation in the present study. Upon analyzing the sgmRNA products of ORF3, we discovered that the YN strain (GAA) produced two transcripts, whereas the mutant strain (TAA) generated only one. Further investigation revealed that the second transcript was generated due to the presence of an additional template-switching site in the YN strain, which caused incomplete pairing between TRS-B and TRS-L at the third position of the CTGAACAA sequence. Thus, it is possible that sgmRNA product diversity can give rise to new variants of known accessory or structural proteins, which may equip viruses with new biological functions or enable them to better adapt to their host environment [59]. This sgmRNA diversity could also account for the observed differences in adaptability between the WT and Δ9aa strains in ECEs and CEK cells. Indeed, similar findings have been reported in other studies. For instance, the IBV Beau-R strain is capable of replicating in a variety of cell lines, including both avian and non-avian cell types, with its ORF3 TRS-B being CTGAACAA. Conversely, the M41-CK strain, which is pathogenic, primarily grows in CEK cells, with its TRS being CTTAACAA [60]. Moreover, the amount of sequence homology between the core sequences of TRS-L and TRS-B correlates positively with sgmRNA abundance [61–63]. Our results also confirmed that the ORF3 genes of rYN-Δ9aa yielded higher levels of sgmRNA transcripts than the corresponding genes of rYN-WT. However, despite clarifying the effect of the TRS-B on the transcription of ORF3 subgenomic transcripts, its impact on IBV virulence warrants further investigation in future studies.

**Fig 9:**
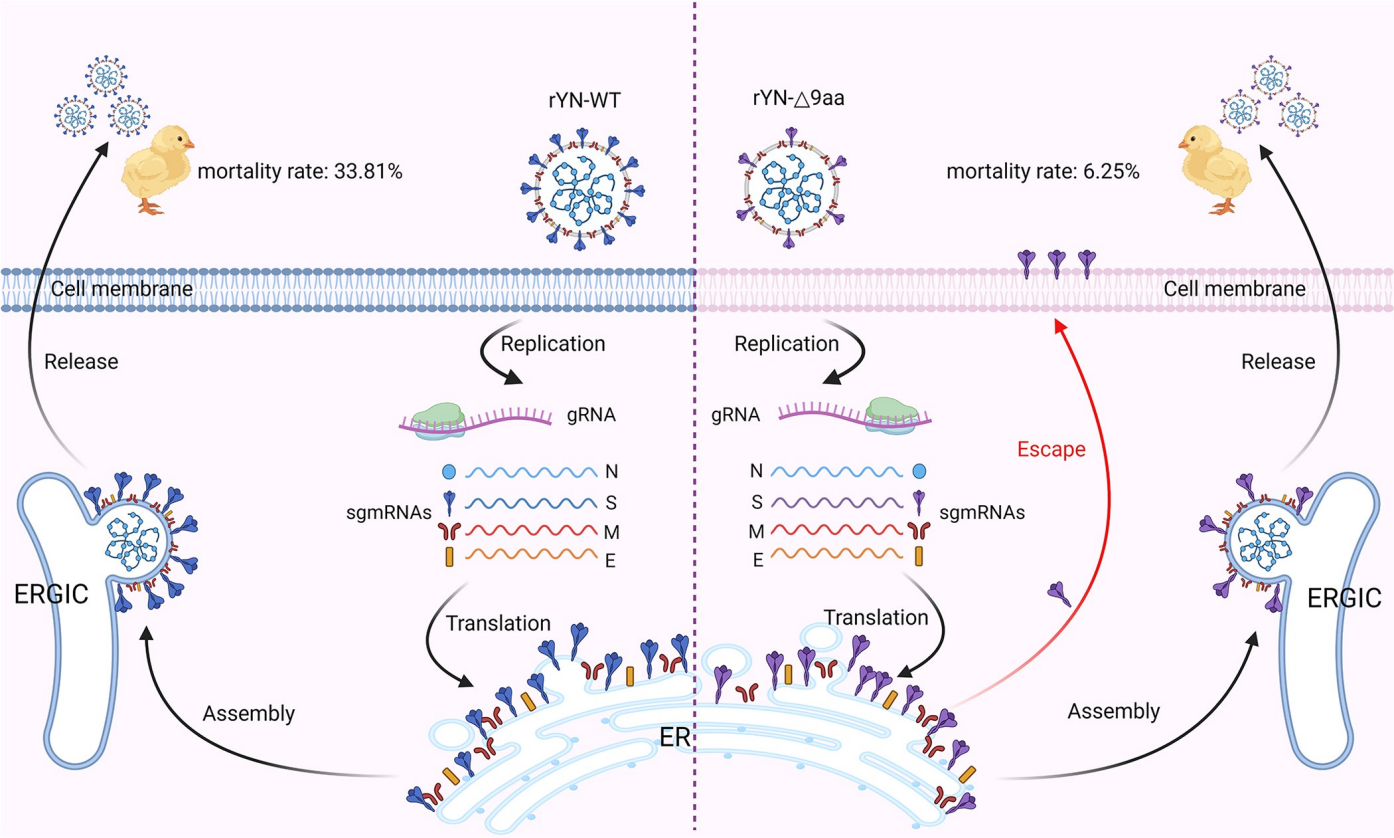
Graphical study summary. The adaptive G to T mutation primarily affects the localization of the S protein, which reduces S protein incorporation into viral particles, subsequently decreasing IBV virulence. This image was created with BioRender.com.

In conclusion, our study elucidated the pivotal role of the adaptive G to T mutation in the CT region of the S gene in the attenuation of IBV virulence. We found that the mutation primarily affected the localization of the S protein and reduced its protein incorporation into viral particles, which ultimately decreases the virus’s invasion efficiency (Fig 9). Furthermore, the favorable adaptation of this mutation to the chicken embryo suggests that this mutation site could serve as a potential target for the development of live attenuated vaccines against IBV. Additionally, similar truncations in the CT of the S protein have been observed in the development of attenuated vaccines for other coronaviruses, such as PEDV. Overall, our findings offer promising, alternative strategies for the development of novel CoV vaccines.

## Materials and methods

### Animals and ethics statement

All SPF ECEs and SPF chickens used in this study were purchased from Beijing Boehringer Ingelheim Vital Biotechnology Co., Ltd. (Beijing, China). The animal experiments were approved by the Animal Welfare and Ethical Censor Committee of China Agricultural University (Approval number: 2023–028).

### Viruses and cells

The QX-like strain YN (GenBank JF893452.2) and the GVI-1 strain NP2011 (GenBank MW815495.1) were preserved in our laboratory, and passaged by inoculation into the allantoic sacs of 10-day-old SPF ECEs. The vaccinia virus vNotI/tk was provided by Dr. Volker Thiel of the University of Bern (Switzerland). The recombinant IBV strains rYN-WT, rYN-Δ9aa, rYN-ΔKKSV, rYN-AKSV, and rYN-KASV were rescued using a previously constructed, vaccinia-virus-based reverse genetic platform [64]. CV-1 and D980R cells were cultured in Minimum Essential Medium (MEM; Thermo Fisher Scientific, Waltham, MA, USA) supplemented with 10% fetal bovine serum (FBS; Gibco, USA). The BHK-21 and HEK-293T cell lines were cultured in DMEM supplemented with 10% FBS and 1% penicillin-streptomycin (Gibco, USA). The CEK cells were isolated from 18-day-old SPF chicken embryos. All cells were grown in a 37°C, 5% CO_2_ humidified incubator.

### Single nucleotide variation analysis of IBV

Total RNA was extracted from cells or the allantoic fluid of chicken embryos infected with IBV and reverse transcribed. Primers were used to amplify the cDNA sequence spanning nucleotides 23,247 to 23,946. To construct the cDNA library, 1 μg of high-quality genomic DNA from each sample was processed using the VAHTS Universal DNA Library Prep Kit for Illumina (Vazyme, ND604), following the manufacturer’s instructions. The genomic DNA was initially fragmented by ultrasonication, before being subjected to end-repair and 3’ end adenylation. Sequencing adapters (Vazyme, N801) were then ligated to the prepared fragments. The ligated fragments were then purified and size-selected using VAHTSTM DNA Clean Beads (Vazyme, N411) to optimize fragment lengths for sequencing. The libraries were amplified by PCR, and the amplified products were subsequently purified. The concentration of the libraries and the size of the inserts were determined using a Qubit 3.0 fluorometer and an Agilent Bioanalyzer 2100 system, respectively. The qPCR was performed on the StepOnePlus Real-Time PCR System (ABI, USA). Indexed samples were clustered using a cBot Cluster Generation System (Illumina, USA) and sequenced on an Illumina NovaSeq 6000 platform by following a 150-bp paired-end protocol. Quality control was performed using Fastp software [65]. The cleaned reads were aligned to the chicken reference genome (Ensembl bGalGal1.mat.broiler.GRCg7b) using hisat2 [66]. Any reads mapping to the host genome were discarded, while the remaining reads were aligned to the reference genomes of IBV strains using the BWA software in MEM mode [67]. PCR duplicates were removed using sambamba [68], and samtools was employed to generate pileup files using the following samtools mpileup commands: -A, -Q 0, -d 100000 [69]. SNV calling was performed using the varscan2 pileup2snp function.

### Generation of mutant viruses

The process for generating infectious, clone-derived IBV mutants has been previously described [64]. In summary, positive selection involved the integration of pGPT-ΔS plasmids into the genome of the vaccinia virus through homologous recombination by replacing the original S gene of rYN with the *gpt* gene in CV-1 cells. After negative selection, the *gpt* gene in the resulting recombinant vaccinia viruses was then replaced with a mutated S gene to generate the rYN-Δ9aa, -ΔKKSV, -AKSV, and -KASV mutants in D980R cells. Capped IBV genomic RNA was transcribed in vitro using the mMESSAGE mMACHINETM T7 transcription kit (Thermo Fisher Scientific, USA). BHK-21 cells were then electroporated with a mixture of full-length rYN-Δ9aa, rYN-ΔKKSV, rYN-AKSV, or rYN-KKSV gRNAs and N gene transcripts. The cells and supernatant underwent three freeze-thaw cycles before being inoculated into 10-day-old SPF ECEs. The recovered viruses were subjected to Sanger sequencing to ensure correct mutation incorporation.

### Viral infection assay

CEK cells were infected with the recombinant IBV strains (i.e., rYN-WT, rYN-Δ9aa, rYN-ΔKKSV, rYN-AKSV, or rYN-KASV) at an MOI of 0.01. Following a 1-hour absorption period at 37°C, the supernatant was discarded, and the cells were washed three times with PBS. The cells were then cultured in DMEM supplemented with 1% FBS, in an incubated at 37°C, 5% CO_2_. Samples were collected at the designated timepoints post-infection.

### Growth kinetics evaluation of infectious recombinant IBVs

Virus growth kinetics were analyzed in both CEK cells and ECEs. The replication of IBV in CEK cells was monitored by inoculating the cells with each of the five recombinant IBVs at an MOI of 0.01. Following a 1-hour absorption period, supernatants were removed, and the cells were washed three times with PBS. Cell samples were then collected at the specified timepoints (1, 6, 12, 24, 36, 48, 60, and 72 hours) for IBV genome detection via RT-qPCR. To monitor viral replication in ECEs, 10^2^ copies of each recombinant IBV were inoculated into 10-day-old ECEs. Allantoic fluids were then harvested at the designated timepoints (12, 18, 24, 36, 48, 60, and 72 hours) and the viral copies was determined using RT-qPCR.

### Quantification of viral adsorption, internalization, and release

To assess viral adsorption, CEK cells were incubated with each viral strain at an MOI of 1 or 10 at 4°C for 1 hour. After ten washes with cold PBS, the relative genome levels were quantified using RT-qPCR. Assessment of viral internalization ability involved the above initial incubation and washing steps. The cells were then rested in DMEM (1% FBS) at 37°C in a 5% CO_2_ incubator for 1 hour, washed ten times with PBS, and treated with protease K (0.5 mg/mL) for 5 minutes to remove surface-adsorbed, non-internalized viral particles. Relative genome levels were again determined by RT-qPCR, with internalization ability defined as the ratio of internalization to adsorption. To assess viral release ability, cell supernatants were collected at 12, 24, and 48 hpi. Subsequently, the cells were washed three times with PBS and lysed. The viral copy number in the supernatant and lysed cells was quantified by RT-qPCR, with release ability defined as the ratio of viral copies in supernatant that in lysed cells.

### Analysis of the relative abundances of sgmRNAs

CEK cells were infected with each of the five recombinant IBVs at an MOI of 0.01. Total RNA was extracted at 12, 24, 36, and 48 hpi and analyzed via RT-qPCR. Primers designed for detecting various lengths of sgmRNAs were used as previously described [70]. The levels of individual sgmRNAs were normalized to those of gmRNA, and their relative abundances were calculated. All sgmRNA detection assays were conducted in triplicate.

### Analysis of IBV protein synthesis

Viral protein synthesis was assessed in CEK cells infected with IBV at an MOI of 0.01. At 24, 36, and 48 hpi, cells were washed with cold PBS and lysed using RIPA buffer (Applygen, Beijing, China). Following a 15-minute incubation on ice, the supernatants were boiled to denature the proteins. Equal amounts of protein were then separated by SDS-PAGE and analyzed via western blotting using specific antibodies against the IBV N protein (Hytest, Turku, Finland), the IBV S2 protein, and β-actin (CST, Danvers, MA, USA). IBV protein signal intensities were normalized against those of β-actin and quantified using ImageJ software.

### Plasmid construction and transfection

The full-length S gene was amplified from YN cDNA. The S-WT, -Δ9aa, -ΔKKSV, -AKSV, and -KASV constructs, with a Flag tag at the S1/S2 cleavage site, were cloned into individual pRK5-Flag vectors. For transfection, cells were seeded on glass coverslips in 12-well clusters. The cells were transfected with each of the above plasmids using the StarFect Transfection Reagent (GenStar, China) according to the manufacturer’s instructions. Briefly, 2 μg of plasmid and 6 μL of StarFect were diluted in 100 μL of Opti-MEM (Gibco) and incubated together for 15 minutes. The cells were then incubated with the plasmid/StarFect complex at 37°C.

### Indirect immunofluorescence assay (IFA) and confocal microscopy

Cell samples were collected at predetermined timepoints post-transfection or -infection and fixed using Immunol Staining Fix Solution (Beyotime Biotechnology, China). This was followed by permeabilization with an Immunostaining Permeabilization Buffer containing Triton X-100 (Beyotime Biotechnology) and subsequent blocking with an Immunol Staining Blocking Buffer (Beyotime Biotechnology). The cells were then incubated with specific primary antibodies at 4°C for 12 hours. For staining, Alexa Fluor 488-conjugated anti-mouse IgG (H+L) and/or Alexa Fluor 555-conjugated anti-rabbit IgG (H+L) (Cell Signaling Technology, USA) were applied at room temperature in the dark for 1 hour. Nuclei were stained with DAPI (Sigma-Aldrich, USA) at room temperature for 10 minutes. Subsequently, the cells were washed five times with PBST (PBS with Tween 20), with each wash lasting 5 minutes. Finally, the cells were observed and imaged using a Nikon A1 fluorescence microscope (Nikon, Tokyo, Japan). Colocalization analysis was performed using the Fiji ImageJ software. PCC is commonly employed to assess the spatial colocalization of two fluorescently labeled molecules or structures within a cell. The PCC values range from -1 to +1, where +1 indicates a perfect positive correlation (complete colocalization), 0 indicates no correlation (random distribution), and -1 indicates a perfect negative correlation.

### Syncytium induction assay

The fusogenic abilities of different Flag-tagged S CT mutants were evaluated in BHK-21 cells. Cells were seeded in 6-well plates and transfected with 2 μg of plasmid DNA encoding each mutant. At 36 hours post-transfection, cells were washed with PBS, fixed in methanol for 30 minutes at room temperature, and stained with Giemsa. The sizes of the syncytia were normalized and quantified using ImageJ software.

### Flow cytometric assessment of cell surface S protein levels

HEK-293T cells were cultured in 6-well plates at 37°C with 5% CO_2_. At 24 hours post-seeding, the cells were transfected with 2 μg of mutant plasmid DNA. At 36 hours post-transfection, the cells were washed three times with ice-cold PBS through resuspension and centrifugation at 500 × g for 5 minutes at 4°C. The supernatant was then discarded, and 10^6^ cells were resuspended in PBS containing a 1:1000 dilution of anti-Flag antibody (CST) and incubated at 4°C for 1 hour. Subsequently, the cells were washed three times with ice-cold PBS and then incubated on ice in the dark for 1 hour with PBS containing FITC-conjugated goat anti-rabbit immunoglobulin G antibodies (diluted 1:500). After three additional washes, the cells were resuspended in 100 μL of cold PBS and strained through a 100-μm filter before being analyzed using a BD FACSCanto II flow cytometer (BD Biosciences). Data were processed using FlowJo software.

### Plasma membrane and cytoplasmic protein extraction

Cells were seeded in a 10-cm culture dish. At 24 hours post-seeding, the cells were transfected with 10 μg of plasmid DNA or infected with IBV at an MOI of 0.01. A cell membrane and cytoplasmic protein extraction kit (Invent) was used to analyze S protein expression at the cell membrane. Briefly, cells were harvested and washed twice with precooled PBS. After discarding the supernatant, cells were resuspended in 500 μL of Buffer A and incubated on ice for 10 minutes. The cell suspension was then transferred to a centrifuge tube column casing and centrifuged at 16,000 × g for 30 seconds. The column was removed, and the sediment was vigorously vortexed, before being centrifuged at 700 × g for 1 minute. The supernatant containing the cytoplasmic fraction was transferred to a new 1.5 mL centrifuge tube and further centrifuged at 16,000 × g for 10 minutes at 4°C. The precipitate was resuspended in 200 μL of Buffer B and centrifuged at 4°C and 7,800 × g for 5 minutes, yielding the organelle fraction. The supernatant was then mixed with 1.6 mL PBS and centrifuged at 16,000 × g for 30 minutes. The final precipitate contained the plasma membrane fraction. The protein composition of the separated fractions (i.e., cytoplasmic, organelle, and plasma membrane) was analyzed by western blotting.

### Transmission electron microscopy (TEM)

To visualize the structure of recombinant IBVs via TEM, 200 mL of culture supernatants were purified. Initially, the supernatant was centrifuged at 4°C and 3,500 × g for 15 minutes, before being layered over a 20% sucrose cushion in an ultracentrifuge tube and centrifuged at 100,000 × g for 2 hours at 4°C. After discarding the supernatant, the pellet was re-suspended in cold PBS. Subsequently, the sample was separated over layers of 30%, 45%, and 60% sucrose, by centrifugation at 100,000 × g for 3 hours at 4°C. The sucrose layer was carefully removed, and the virus band at the 45% sucrose interface was collected and transferred to a new tube. The virus was diluted with PBS and centrifuged for 3 hours at 100,000 × g and 4°C to remove the sucrose. The supernatant was discarded, and the purified viral pellet was re-suspended in 4% paraformaldehyde. The fixed samples were then applied to a carbon-coated copper grid, negatively stained with 1% phosphotungstic acid (pH 7.0), and examined under a transmission electron microscope.

### Animal experiments

SPF chickens were randomly allocated into six groups and housed in separate isolators. Each group was inoculated with 100 μL of rYN-WT, rYN-Δ9aa, rYN-ΔKKSV, rYN-AKSV, rYN-KASV (each at 10^5^ EID_50_), or PBS, administered as eye drops. Clinical signs such as sneezing, tracheal rales, and somnolence were monitored and documented until 14 dpc. At 3, 5, 7, and 14 dpc, three chickens from each group were euthanized and subjected to necropsy. Presence of gross lesions in the trachea, lungs, and kidneys were recorded. The tissues of these organs were also evaluated for viral presence using RT-qPCR and subjected to histopathological examination. The severity of the lesions was evaluated using predefined scoring criteria [71]. The tracheal ciliary activity and mean lesion scores were assessed as previously described [72].

### Leader-body junction RT-PCR analysis

cDNA from the IBV YN strain served as the template for amplifying each sgmRNA and identifying its CSs. Amplification of each subgenome was performed using the Taq polymerase KOD One PCR Master Mix-Blue (TOYOBO, Japan), with primers specific to the leader sequence and the respective sgmRNAs described in the literature [70]. The PCR products were cloned using the T-Vector pMD19 (Simple)+ DNA Ligation Kit Ver.2.1 (TAKARA, Japan) and then transformed into DH5α competent cells. Single-clone colonies were then selected for sequencing analysis.

### Quantification of IBV sgmRNAs by strand-specific RNA-seq

CEK cells were inoculated with PBS, rYN-WT, or rYN-Δ9aa, with three replicate samples in each treatment group. At 24 hours post-inoculation, the cells were collected for RNA extraction. The quality of the extracted RNA was evaluated using 1% agarose gels, while the RNA purity and concentration determined using a NanoPhotometer and a Qubit 2.0 Fluorometer, respectively. In addition, RNA integrity assessed using an Agilent Bioanalyzer 2100 system. For strand-specific RNA sequencing, 1.5 μg of RNA was prepared using the NEBNext Ultra Directional RNA Library Prep Kit for Illumina, by following the manufacturer’s instructions and using index codes for sample identification. mRNA isolation involved poly-T oligo-attached magnetic beads, with fragmentation induced by divalent cations at elevated temperatures. cDNA synthesis utilized random hexamer primers, with M-MuLV Reverse Transcriptase for the first strand and DNA Polymerase I and RNase H for the second, substituting dTTP with dUTP. Amplification with USER Enzyme and PCR were performed following end repair, adaptor ligation, and size selection. Libraries were purified and quality-checked on an Agilent Bioanalyzer 2100 system, clustered using a cBot Cluster Generation System and HiSeq 4000 PE Cluster Kit (Illumina), and sequenced on an Illumina HiSeq 4000 platform, generating 150-bp paired-end reads.

Initial quality control of raw sequencing reads was performed using Fastp software [65]. This step involved removing low-quality reads and trimming the first 15 bp from each read. The clean reads were then aligned to the chicken reference genome (Ensembl bGalGal1.mat.broiler.GRCg7b) using hisat2 [66]. Reads mapping to the host genome were discarded. The remaining reads were aligned separately to the reference genomes of the IBV YN strain (GenBank JF893452.2) and its mutant strains with STAR [73] using the following parameters: STAR—genomeDir $GENOME_DIR—runThreadN 16—readFilesIn $read1 $read2—readFilesCommand zcat—outFileNamePrefix $PREFIX—outSAMtype BAM SortedByCoordinate—outSAMattributes All—outFilterType BySJout—outFilterMultimapNmax 20—alignSJoverhangMin 8—alignSJDBoverhangMin 1—outSJfilterOverhangMin 12 12 12 12—outSJfilterCountUniqueMin 1 1 1 1—outSJfilterCountTotalMin 1 1 1 1—outSJfilterDistToOtherSJmin 0 0 0 0—outFilterMismatchNmax 999—outFilterMismatchNoverReadLmax 0.04—scoreGapNoncan 0—scoreGapGCAG 0—scoreGapATAC 0—chimOutType Junctions WithinBAM HardClip—chimScoreJunctionNonGTAG 0—alignSJstitchMismatchNmax -1–1–1–1—alignIntronMin 20—alignIntronMax 1000000—alignMatesGapMax 1000000—chimSegmentMin 20—outSAMmultNmax 32—limitBAMsortRAM 1187802886. The IBV sgmRNAs were defined based on the junction read splice sites (Tables 1 and 2). The expression levels of sgmRNAs were normalized against the total number of viral-genome-mapped reads in each sample.

### Statistical analysis

All data were analyzed with GraphPad Prism software version 5.0 (GraphPad Software Inc., San Diego, CA, USA). Student’s t-test was used to determine the significance of differences between two groups, while one-way and two-way analyses of variance (ANOVA) were for multiple group comparisons. *P*-values < 0.05 were used a measure of statistical significance, with ns, not significant; *, *P* < 0.05; **, *P* < 0.01; ***, *P* < 0.001 indicated in the figure captions.

## Supporting information

S1 Table Mutation sites in the IBV YN after 100 passages in chicken embryos. (PDF)

S2 Table Mutation sites in the IBV NP2011 after 100 passages in chicken embryos. (PDF)

S1 Fig Quantify the transcription levels of sgmRNAs by strand-specific RNA-seq. (A) Relative junction-spanning reads of S. (B) Relative junction-spanning reads of M. (C) Relative junction-spanning reads of N. (D) Relative junction-spanning reads of ORF5.
(TIF)

S1 Data Datasheet containing the raw data and original uncropped pictures. (XLSX)
