# Supplementary material for: Adaptive truncation of the S gene in IBV during chicken embryo passaging plays a crucial role in its attenuation
Source: PLoS Pathog. 2024 Jul 30;20(7):e1012415. doi: 10.1371/journal.ppat.1012415 (PMC11315334; doi:10.1371/journal.ppat.1012415)
Supplement: S1 Table — (PDF) [file ppat.1012415.s001.pdf]

S1 Table. Mutation sites of IBV YN after 100 passages in chicken embryos.

sites	Nucleotide difference	Amino acid difference	Gene region
225	C→G	-	5’UTR
808	A→C	K→T	1a
2764	C→T	P→L	1a
2899	C→A	A→D	1a
3369	G→T	V→F	1a
3693	G→T	D→Y	1a
5815	A→G	N→S	1a
7059	G→C	D→H	1a
7359	T→C	W→R	1a
9438	G→T	D→Y	1a
9490	G→T	S→I	1a
13131	C→T	S→F	1a
13284	C→T	S→F	1a
13457	G→C	G→R	1a
14547	C→T	A→V	1a
14718	C→G	S→C	1a
19659	C→T	S→L	1a
20455	T→C	V→A	S
20515	C→T	T→I	S
20646	A→G	M→V	S
20659	A→G	K→R	S
20722	T→C	F→S	S
20757	G→A	E→K	S
21370	G→A	R→K	S
21543	C→G	R→G	S
22434	C→T	P→S	S
22842	T→G	Y→D	S
23835	G→T	E→*	S
25527-25607	81nt deleted	27 aa deleted	5a
26519	G→T	R→L	N
26908	A→C	T→P	N
26987	C→T	P→L	N
