# Supplementary material for: Adaptive truncation of the S gene in IBV during chicken embryo passaging plays a crucial role in its attenuation
Source: PLoS Pathog. 2024 Jul 30;20(7):e1012415. doi: 10.1371/journal.ppat.1012415 (PMC11315334; doi:10.1371/journal.ppat.1012415)
Supplement: S2 Table — (PDF) [file ppat.1012415.s002.pdf]

S2 Table. Mutation sites of IBV NP2011 after 100 passages in chicken embryos.

sites	Nucleotide difference	Amino acid difference	Gene region
1266	C→T	A→V	1a
1308	C→T	P→L	1a
1840	A→C	K→N	1a
2848	T→A	D→E	1a
3345	C→T	A→V	1a
6304	C→T	-	1a
7320	C→T	T→I	1a
8331	C→T	A→V	1a
10184	C→T	L→F	1a
10248	C→T	A→V	1a
12290	G→A	A→T	1a
17460	G→A	R→K	1a
20406	C→T	S→L	S
20639	A→T	N→Y	S
20670	A→C	K→T	S
21198	T→C	I→T	S
21930-21933	3nt deleted	1 aa deleted	S
22768	A→G	-	S
22795	A→G	-	S
23365	C→T	-	S
23678	G→T	G→C	S
23774	G→T	E→*	S
24226	C→T	-	E
24858	G→T	S→I	M
25122	C→T	T→I	M
25453	1nt insert		4b
25729	A→G	N→S	5b
26607	G→T	G→C	N
26671	C→T	T→I	N
26742	C→T	P→S	N
