# Supplementary figures and images for: Adaptive truncation of the S gene in IBV during chicken embryo passaging plays a crucial role in its attenuation
Source: PLoS Pathog. 2024 Jul 30;20(7):e1012415. doi: 10.1371/journal.ppat.1012415 (PMC11315334; doi:10.1371/journal.ppat.1012415)

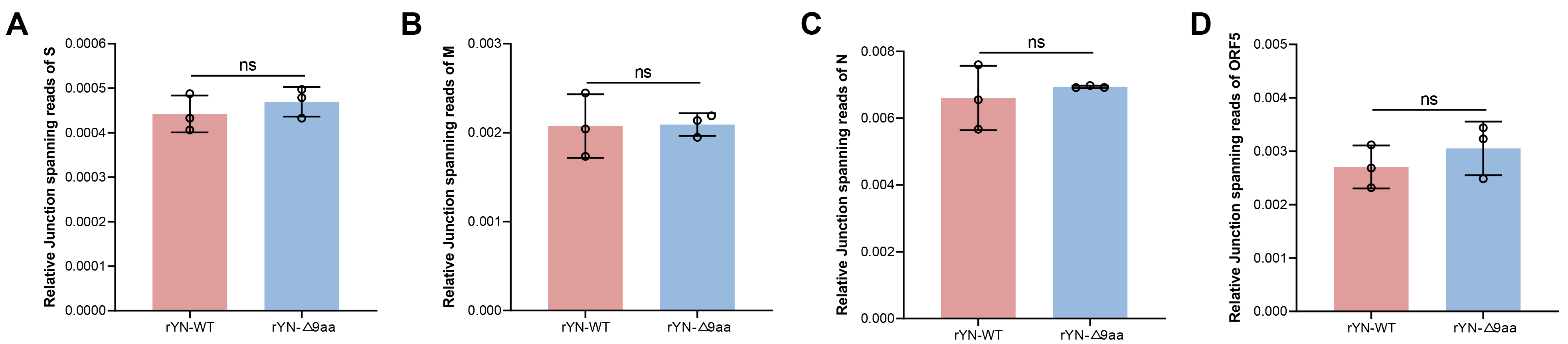

Supplement: S1 Fig — (A) Relative junction-spanning reads of S. (B) Relative junction-spanning reads of M. (C) Relative junction-spanning reads of N. (D) Relative junction-spanning reads of ORF5. (TIF) [file ppat.1012415.s003.tif]
